# Preoperative diagnosis of primary ovarian lymphoma: a case report and a decade of insights

**DOI:** 10.3389/fonc.2024.1471654

**Published:** 2024-12-11

**Authors:** Wei Liu, Houyun Xu, Jibo Hu, Xiping Yu, Junjie Zhou, Hongjie Hu

**Affiliations:** ^1^ Department of Radiology, the Fourth Affiliated Hospital of School of Medicine, and International School of Medicine, International Institutes of Medicine, Zhejiang University, Yiwu, China; ^2^ Department of Pathology, the Fourth Affiliated Hospital of School of Medicine, and International School of Medicine, International Institutes of Medicine, Zhejiang University, Yiwu, China; ^3^ Department of Radiology, Sir Run Run Shaw Hospital, Zhejiang University School of Medicine, Hangzhou, Zhejiang, China; ^4^ Medical Imaging International Scientific and Technological Cooperation Base of Zhejiang Province, Hangzhou, China

**Keywords:** primary ovarian lymphoma, imaging features, diffuse large B cell lymphoma, hematologic malignant, diagnosis

## Abstract

Through a comprehensive retrospective analysis of a 52-year-old woman with primary ovarian lymphoma (POL) and a review of similar cases over the past decade in the PubMed database, we gained several key insights into improving the understanding of POL among clinicians and radiologists for accurate diagnosis. POL is more prevalent among women in their 40s and usually presents with clinical manifestations of a solid mass (typically over 10 cm) and abdominal pain with B symptoms. Four imaging features show promise as indicators of potential diagnostic value in POL: the ovarian retention sign, touching ovaries, vascular floatation, and the sandwich sign. More than half of primary ovarian diffuse large B-cell lymphoma (DLBCL) cases have elevated lactate dehydrogenase (LDH) or carbohydrate antigen 125 (CA-125) levels. This comprehensive understanding of POL suggests that the combination of these four imaging features with elevated levels of CA-125 and LDH might help in the diagnosis of POL preoperatively, preventing unnecessary surgical interventions.

## 1 Background

Genital tract involvement in primary extra-nodal non-Hodgkin lymphoma (NHL) is rare, only accounting for 0.5% of all NHLs (1). Primary ovarian lymphoma (POL) has a lower incidence. Due to its non-specific clinical presentations and the absence of thorough or inaccurate radiological assessments, there might be potential delays in its diagnosis. This study aimed to improve the understanding of POL by delving into the subtle imaging characteristics of this case and by reviewing similar cases documented in the literature.

## 2 Case representation

On September 8, 2022, a 52-year-old woman presented at our hospital complaining of an abdominal mass and intermittent abdominal pain since July 2022. Initially, the palpable mass, which was as large as an egg, was in the left lower abdomen. Without any treatment, the mass showed continuous growth, ultimately expanding into the entire lower abdomen. The patient denied any additional symptoms, such as fever, vomiting, or hematochezia. Throughout the course of her illness, the patient had lost approximately 5 kg in weight. A review of the laboratory examination of this patient revealed relevant abnormal indicators, as shown in **Table 1**.

**Table 1:** Review of the laboratory test data of the present case.

	Preoperative	Immediate postoperative	F/U 6 months	F/U 12 months	Reference
CA-125 (U/ml)	198.04↑	–	9.18	9	<24.0
CYFRA21-1 (ng/ml)	2.37↑	–	1.84	7.3	<2.08
NSE (ng/ml)	82.88↑	–	15.09	12.55	<25.00
LDH (U/L)	1,800↑	328↑	191	160	120–250

CA-125, carbohydrate antigen 125; CYFRA21-1, cytokeratins 19 fragment antigen21-1; NSE, neuron specific enolase; LDH, lactate dehydrogenase; F/U, follow-up. ↑, higher than reference value.

**Figure 1:**
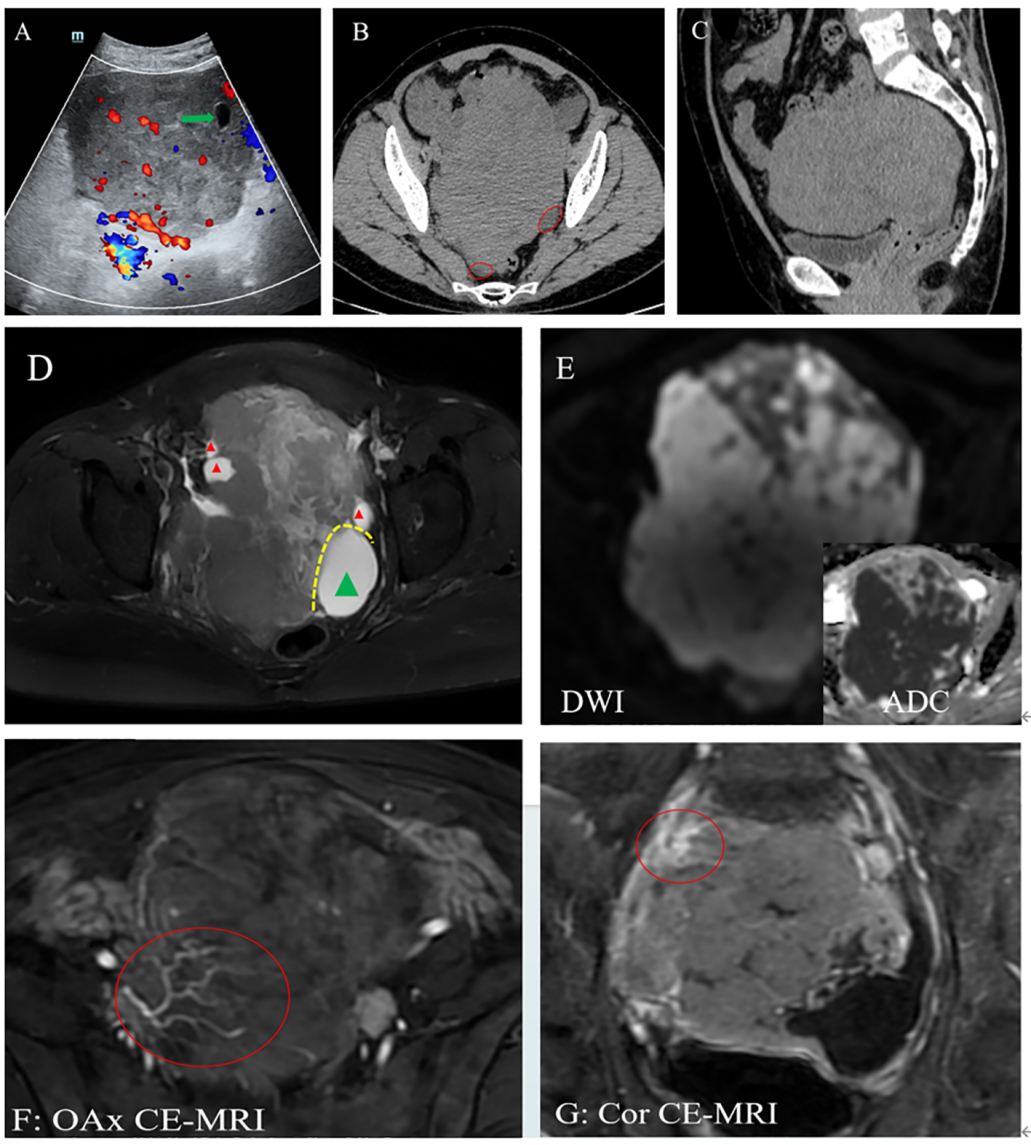
**(A)** Ultrasound (US): A hypoechoic lesion in the pelvis with a clear boundary and a regular shape, with blood flow signal. A cystic area was at the edge of the lesion (*green arrow*). **(B, C)** Computed tomography (CT): A heterogeneous hypodense mass with ascites (*red circle*). The CT value was approximately 40 HU. The adjacent bowels and urinary bladder were pushed. **(D–G)** Magnetic resonance imaging (MRI): Plain scan showing the right ovary as enlarged with multiple peripheral follicles seen at the edge on T2-weighted imaging (T2WI) (*red triangle*). **(D)** Left ovary showing cystic enlargement (*green triangle*). These two ovaries are in contact with each other due to enlargement. **(E)** On diffusion-weighted imaging (DWI), the mass presented as a heterogeneous high signal, and its solid component had a low apparent diffusion coefficient (ADC) value. **(F, G)** Enhanced image showing vascular floatation sign **(F)** and the sandwich sign **(G)**, exhibiting that normal blood vessels walked within the tumor and the right ovarian vein was encased by the tumor, respectively.

For further aid in diagnosis, the patient subsequently underwent comprehensive imaging examinations. Ultrasound (US) suggested a hypoechoic solid mass in the pelvic cavity measuring 14 × 10 × 11 cm (**Figure 1A**). A plain CT scan of the abdomen revealed a heterogeneous hypodense mass with ascites (**Figures 1B, C**). In the abdominal pelvic MRI, the mass was considered to originate from the right ovary, displaying mixed high signal on T2-weighted imaging (T2WI) (**Figure 1D**) and a hypointense signal on T1-weighted imaging (T1WI). The mass had a heterogeneous high signal on diffusion-weighted imaging (DWI) (**Figure 1E**). Enhanced MRI showed the tumor artery originating from the internal iliac artery, which exhibited moderate delayed enhancement (**Figure 1F**). Moreover, coronal MRI showed the tumor encasing the right ovarian vein (**Figure 1G**).

Lymphadenopathy or splenomegaly was not noted. The chest CT did not show obvious abnormalities. We first suspected ovarian fibroma.

Subsequently, the patient underwent a bilateral salpingo-oophorectomy on September 12, 2022. The surgical procedure disclosed a 14-cm mass originating from the right ovary, adherent to the right bladder and pelvic wall. The enlarged left ovary was cystic. The intraoperative pathology suggested “malignant tumor, high grade,” leading to the expansion of the surgical scope. These resected tissues were then pathologically examined. The immunohistochemistry (IHC) results of the tumor tissues in the right ovary revealed positive expression of CD19, CD20, CD45, CD79a, Bcl-6, Bcl-2, and MUM-1. The positivity rate of Ki-67 was over 90%. Notably, no tumor cells were detected in the fallopian tube and the left ovary. The timeline of this patient is shown in **Supplementary Figure S1**.

Postoperative positron emission tomography (PET) showed that only the right iliac lymph nodes had pathological glucose metabolism pattern. Bone marrow biopsy showed no evidence of malignant cells.

Based on the IHC results and Han’s classification, the case was definitively diagnosed as diffuse large B-cell lymphoma (DLBCL), non-GCB (germinal center B cell) subtype, at stage IIB.

The patient was then treated with R-CHOP (rituximab, cyclophosphamide, doxorubicin, vincristine, and prednisone) chemotherapy, resulting in complete remission. She is currently being monitored with serial PET scans and continues to be in remission after 24 months without any evidence of relapse.

## 3 Discussion

To our knowledge, this case, as a representative instance of POL, is the first report with comprehensive imaging examinations, encompassing four distinctive imaging manifestations. Until now, there has been a lack of consensus regarding the tissue origin and pathogenesis of POL. When a space-occupying lesion appears in the ovary, the mass is rarely considered as lymphoma, particularly POL. However, in their investigation of 37 oophorectomy specimens, Skodras et al. confirmed the presence of scattered lymphocytes and small lymphoid aggregates through IHC analysis (2, 3).

The first and foremost step in the diagnosis of POL is to accurately identify whether the observed ovarian lesion is indeed a lymphoma. Due to its atypical site of origin, non-specific clinical manifestations, and the lack of comprehensive or accurate radiological evaluations, the diagnosis of POL has previously relied on postoperative pathological findings (3). In this situation, these may potentially lead to an expanded range of surgical resection (as in the present case) or a delay in diagnosis (4–6). If radiologists can identify the four characteristic imaging manifestations shown in our case and suggest the preoperative possibility of ovarian lymphoma, imaging examinations, as routine noninvasive preoperative diagnostic approaches, could guide clinicians in providing personalized treatment for patients and in improving patient prognosis.

One of the key points is a so-called ovarian retention sign. This manifestation refers to the presence of scattered cystic structures, namely, follicles, observed at the edges of the ovary. As demonstrated in the research by Kim et al. (4), the preservation of peripheral follicles is significantly more common in ovarian lymphomas compared with other solid ovarian tumors. This phenomenon suggests the presence of ovarian lymphomas (7), as ovarian lymphomas preserve some normal structures of the ovarian tissue during their growth, while other solid ovarian tumors typically destroy and replace the normal ovarian tissue structure.

The “touching ovaries” sign is also an indication, denoting a physical contact between the bilateral ovaries due to their enlargement with loss of fat planes between them (8). This phenomenon is particularly evident when both ovaries are involved. However, ovarian endometriosis can show a similar finding, known as “kissing ovaries.” Unlike the affected ovaries in POL, which are typically solid and well circumscribed, ovarian endometriomas are often multilocular and are accompanied by inter- or peri-ovarian adhesions (9). Moreover, the abdominal pain associated with ovarian endometriomas is correlated with the menstrual cycle.

Another sign is vascular floatation, which refers to normal-shaped blood vessels traversing within the interior of a tumor. This characteristic can also be observed in lymphomas occurring in other anatomical locations (10). Vascular floatation serves as a unique indicator of lymphomas, and when the site of the lesion is confirmed, the possibility of an ovarian lymphoma should be considered.

The sandwich sign, also known as the hamburger sign, is a critical manifestation in the diagnosis of lymphoma. This concept was first described in 1976 as a specific finding to mesenteric lymphoma (11). In 2014, Chien (7) applied this concept to ovarian lymphoma, demonstrating that the tumor encases the artery and the vein as they pass through the hilum and extend upward along the gonadal vessels. This feature is clearly identifiable on coronal imaging.

When a solid tumor is present in the ovary, it is crucial to carefully identify the aforementioned four subtle imaging signs, as the optimal management of ovarian lymphoma involves a combination of tissue confirmation through biopsy and appropriately tailored chemotherapy (4). If the possibility of lymphoma can be considered preoperatively, it may be feasible to avoid excessive surgical treatment.

The second step in the diagnosis of POL is to differentiate it from the secondary ovarian lymphoma. POL exhibits a higher 5-year survival rate (approximately 70%–80%) than secondary lymphoma (12, 13). One principal criterion is that, at the moment of diagnosis, the lymphoma is confined to the ovary and investigation reveals no lymphomatous localization elsewhere. Moreover, if an adjacent lymph node or structure is involved, POL can also be considered (12). PET-CT, combined thoracic and abdominal CT or MR scans, and bone marrow biopsies are invaluable tools in determining the stage of the disease, which were also used in this case.

To achieve a thorough comprehension of POL from diverse perspectives, we searched for similar cases in the last decade, focusing solely on the English literature available in the PubMed database. The exclusive criteria encompassed cases devoid of pathological evidence from bone marrow biopsy or lacking imaging findings indicative of POL (*n* = 5), as well as cases where the subtypes were not DLBCL (*n* = 9). Ultimately, a total of 23 cases were analyzed (**Table 2**; **Supplementary Table S1**). All statistical analyses were conducted on a case-by-case basis. Continuous variables were compared using Student’s *t*-test, while categorical variables were compared among different groups using the *χ* ^2^ test or Fisher’s exact test. All statistical analyses were conducted using SPSS 26.0, and a *p*-value <0.05 was considered statistically significant.

**Table 2:** Clinical characteristics of the 23 patients.

Characteristics	Total (*n* = 23)
Age (years) ^a^	43.0 ± 20.0
Symptom ^a^	
Abdominal pain	15 (65.2)
Abdominal distension	9 (39.1)
B symptoms	16 (69.6)
Diameter (cm) ^a^	12.8 ± 5.1
Diameter >10 cm ^a^	14 (60.9)
Site	
Left	11 (47.8)
Right	9 (39.1)
Bilateral	3 (13.0)
Elevated level of LDH ^b^	10 (43.5)
Elevated level of CA-125 ^c^	12 (52.2)
Subtype (*n*) ^d^	
GCB	7 (30.4)
Non-GCB	7 (30.4)
Survival (months) ^e^	14.4 ± 10.22

^a^Missing for one patient. ^b^Missing for eight patients. ^c^Missing for five patients. ^d^Missing for nine patients. ^e^Missing for eight patients

In our analysis, the age of the patients ranged from 5 to 75 years, with a mean of 43.0 years. The most prevalent symptom was abdominal pain (*n* = 15, 65.2%), followed by abdominal distension (*n* = 9). There were 16 patients (69.6%) who had B symptoms, including fever, night sweats, fatigue, and weight loss. B symptoms have been frequently reported in ovarian lymphomas, and in our research, the rate was even higher than that previously reported (ranging from 10% to 33%) (14, 15). Details of the clinical symptoms are displayed in **Supplementary Table S1**. The affected ovaries were enlarged to various degrees, ranging from 5 to 22 cm, but the majority of these cases were over 10 cm. Most of the cases were unilateral (87.0%), with 11 cases involving the left and nine cases involving the right ovary. Only three cases were bilateral, contrary to previous research reports that described involvement of both ovaries being common and the right ovary being more susceptible than the left (14, 16).

Of the total 23 patients, eight patients did not have data on the LDH level. Among the remaining 15 patients, 10 exhibited elevated levels of LDH. The CA-125 level was elevated in 12 patients, while five patients did not have these data recorded. When combined with previous research, it could be inferred that POL may often be accompanied by elevated levels of LDH and CA-125 (17). Several studies have reported that LDH and CA-125 are correlated with the staging of patients, with higher levels of LDH and CA-125 in patients with stage III–IV lymphoma than in early-stage patients (18, 19). However, the levels of LDH and CA-125 showed no statistical significance between the different stages in our research (*p* = 1.000 and 0.094, respectively), as shown in **Supplementary Table S2**. This could be due to the limited sample size and the high rates of missing data. In the future, a larger sample size is necessary to strengthen the robustness of the results.

It has been reported that the worse prognosis of POL may be related to bilateral ovarian involvement, elevated LDH and CA-125 levels, and the advanced stage or the DLBCL subtype (2, 14, 20–22). Limited-stage DLBCL (conventionally defined as Ann Arbor stages I–II) is excellent, with a 10-year overall survival of at least 70%–80% (23). As illustrated in the aforementioned cases, with the exception of two patients who died definitively (one due to COVID-19), no deaths were reported among the remaining patients, and 15 patients were disease-free during follow-up, including patients at stage IV (*n* = 6). There was no statistical difference in any of the clinical characteristics between the early and advanced stages (**Supplementary Table S2**). It is likely that patients with primary ovarian DLBCL may have good prognosis regardless of the levels of LDH and CA-125 and the size, site, or stage of the tumor. A probable reason may be related to the tumor staging criteria: in four cases, the POL at stage IV was confined to bilateral ovaries without distant metastasis or involvement of, e.g., the bone marrow or the liver. A larger sample size is needed to verify this conclusion. In addition, while most of the cases have undergone surgical resection and chemotherapy, two cases achieved complete remission solely through chemotherapy.

POL presents with common symptoms such as abdominal pain, distension, and ascites and can mimic other gynecologic entities (24), presenting a challenge in accurate diagnosis. Unlike other ovarian neoplasms that typically require surgical intervention, POL can be effectively treated with chemotherapy (3). It is crucial to differentiate POL from other ovarian solid neoplasms. In instances of bilateral ovarian tumors, it is imperative to distinguish POL from metastatic carcinomas by judiciously integrating the patient’s clinical history. When tumors occur unilaterally in the ovaries, POL needs to be distinguished from other ovarian neoplasms, such as fibroma, fibrothecoma, and dysgerminoma, which typically do not exhibit peripheral cysts in the affected ovaries. Fibroma and fibrothecoma have low signal intensity on T2WI due to their low water content, which is easily distinguished from lymphomas (4). Dysgerminoma commonly occurs in younger individuals, and most lesions exhibit characteristic fibrovascular septa (25). Although epithelial ovarian carcinomas comprise the most common type of malignant ovarian tumors, they predominantly present as cystic or cystic–solid masses, with substantial enhancement in the solid areas. Therefore, when solid tumors involve ovaries, the likelihood of an epithelial ovarian carcinoma is comparatively low.

## 4 Conclusion

In general, we recognized four imaging features, namely, the ovarian retention sign, touching ovaries, vascular floatation, and the sandwich sign, of the POL in our case. Through a review of similar cases in the last decade, we found that the majority of patients with primary ovarian DLBCL had elevated LDH and CA-125 levels. For clinicians and radiologists, it is feasible to consider the possibility of POL preoperatively by combining subtle imaging signs with elevated levels of CA-125 and LDH, preventing excessive surgical interventions. However, due to the limited sample size, these findings still need to be verified in the future.

## Data Availability

The original contributions presented in the study are included in the article/**Supplementary Material**. Further inquiries can be directed to the corresponding authors.
