# Supplementary material for: Preoperative diagnosis of primary ovarian lymphoma: a case report and a decade of insights
Source: Front Oncol. 2024 Dec 11;14:1471654. doi: 10.3389/fonc.2024.1471654 (PMC11668800; doi:10.3389/fonc.2024.1471654)
Supplement: Supplementary file 1 [file DataSheet1.pdf]

frontiers

Supplementary Material

1 Supplementary Figures and Tables

1.1 Supplementary Figures

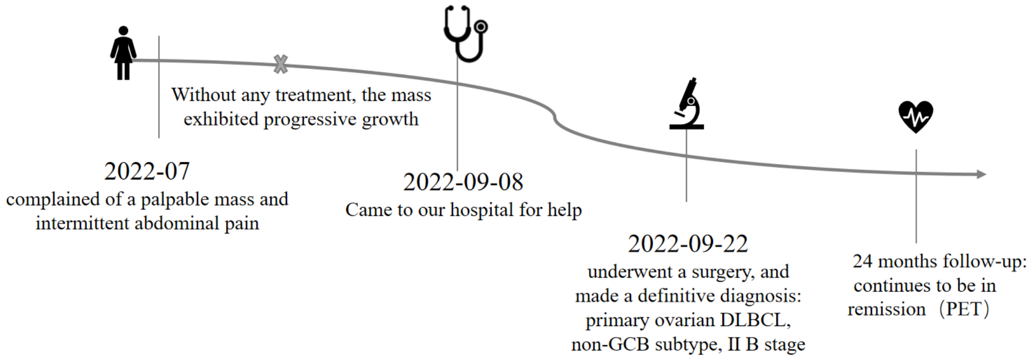

Figure S1. Timeline of this case.

1.2 Supplementary Tables

Table S1. Summary of primary ovarian lymphoma over the past decade reported cases.

Cases	Age	Clinical manifestations	B symptom	diameter (cm) and side	Abnormal laboratory indicator	Treatment	Histologic type and Ann Arbor stage	Abnormal indicating imaging features	Immunophenotype	Survival or follow-up time (months)
1(1)	61y	abdominal distension	fatigue	20, right	CA-125: 312 U/mL↑; LDH: 417 IU/L↑	Surgery, chemotherapy	DLBCL, I B	US/CT: non	CD20(+), CD45(+), Ki-67(+), bcl-2(+)	20
2(1)	52y	abdominal distension	fatigue	16, right	CA-125: 41 U/mL↑; LDH: normal	Biopsy, chemotherapy	DLBCL, I B	CT: non	CD20(+), bcl-2(+)	NED, 12
3(1)	57y	abdominal distension	fatigue, night sweets	16, left	CA-125: 220 U/mL↑; LDH: 2012 IU/L↑	Biopsy	DLBCL, I B	CT: non	CD20(+), bcl-2(+)	1
4(2)	45y	nausea, vomiting, abdominal discomfort	weight loss	16, right; 6, left	CA-125: 1366 U/mL↑; LDH: NM	Surgery, chemotherapy	DLBCL, IV B	CT: ①peripheral cysts in involved ovaries, ②tumor encasing the hilar vessels.	NM	NED, 12
5(3)	52y	abdominal pain and distension	fatigue	16, left	CA-125 and LDH: normal	Surgery, chemotherapy	DLBCL, I B	US/CT: non	CD20(+), LCA (+)	NED, 12
6(4)	50y	abdominal pain	fever, night sweets	15, left	CA-125: normal; LDH: NM	Surgery, chemotherapy	DLBCL, (GCB), I B	US/CT: non	CD10(+), CD20(+), bcl-2(+), bcl-6(+)	NED, 24
7(5)	37y	anemia, abdominal distension	No	11, right; 7, left	CA-125: 766 U/mL↑; LDH: 654 U/L↑	Surgery, chemotherapy	DLBCL, IV	CT/PET-CT: non	CD43(+), CD56(+), CD79(+), bcl-2(+); CD20(-)	NED, 18
8(6)	15y	abdominal pain, hypertension	No	22, right; 16, left	CA-125：934.8 U/ml↑; LDH: 1245 U/L↑	Surgery	DLBCL(GCB), IV	US/MR: non	CD19(+), CD20(+), CD22(+), Ki-67(+) =90~95%, PAX-5(+), bcl-6(+)	NM

9(6)	5y	dysuria, abdominal distension	fever	6, right	CA-125: normal; LDH: 255 U/L↑	Biopsy, chemotherapy	DLBCL (non-GCB), I B	CT: non	CD19(+), CD20(+), CD22(+), CD43(+), Ki-67(+) =90%, bcl-2(+), bcl-6(+)	NED, 24
10(7)	30y	abdominal pain	fatigue, fever	11, left	CA-125: NM; LDH: 878 U/L↑; HIV+	Surgery	DLBCL (GCB), I B	CT: non	CD20(+), bcl-6(+), C-Myc(+), Ki-67(+)>90%, CD10(-), MUM-1(-)	NM
11(8)	34y	abdominal pain and distension	fever, night sweets	20, left	CA-125：44U/mL↑; LDH: NM; HIV+	Surgery, chemotherapy	DLBCL, IV B	US: non	NM	NED, 1
12(9)	21y	abdominal pain and distension, constipation, anemia	No	18, left	CA-125: 27 IU/mL; LDH: NM	Surgery, chemotherapy	DLBCL, (GCB), I	US/CT: non	CD20(+), CD45(+), Ki-67(+) =100%, bcl-2(+), bcl-6(+), C-Myc(+), MUM-1(-), CD10(-)	NED, NM
13(10)	73y	Non	fever, fatigue	7, left	CA 125: NM; LDH: 517 IU/L↑	Surgery, chemotherapy	DLBCL, I B	MR: non；PET-CT: pathological glucose metabolism	CD20(+)	NED, NM
14(11)	59y	abdominal pain	fever	7, right	CA-125: NM; LDH: 2891 U/L↑	Laparoscopic Surgery, chemotherapy	DLBCL, III B	US/CT: non	CD3(+), CD20(+), Ki-67(+) =30~40%	NED, 6
15(12)	29y	abdominal pain	No	8, left	CA199: 65.77 IU/mL↑; CA-125: 9.1 IU/mL; LDH: NM	Laparoscopic Surgery, chemotherapy	DLBCL (non-GCB), II	US/CT: non	CD3(+), CD5(+), CD20(+), Ki-67(+)>90%, bcl-6(+), MUM-1(+), CD10(-)	NED, NM
16(13)	NM	NM	No	NM, right	NM	NM	DLBCL (non-GCB), III	NM	CD20(+), CD79a (+), Ki-67(+) =90%, bcl-6(+), MUM-1(+), CD10(-)	NM
17(14)	11y	abdominal pain	weight loss	15, right	CA-125: 134 IU/L↑; LDH: 526 U/L↑	Surgery	DLBCL (GCB), I B	US/CT: non	CD10(+), CD20(+), CD45(+), Ki-67(+) =80~90%, TdT(+)	NM
18(15)	65y	abdominal pain	fever	9, left	CA-125: normal; LDH: NM	Surgery, chemotherapy	DLBCL (GCB), II B	US: non；PET-CT: pathological glucose metabolism	CD20(+), CD30(+), Ki-67(+) =90%, bcl-2(+), bcl-6(+), C-Myc(+), CD10(-), MUM-1(-)	NED, 5

19(16)	75y	abdominal pain, bowel habits change	weight loss	5, right	Normal	Surgery	DLBCL (non-GCB), IV B	CT:	CD20(+), CD43(+), CD79a(+), PAX-5(+), bcl-6(+), MUM-1(+), P53(20%), Ki-67(80%)	NM
20(17)	55y	abdominal pain	No	8, right	CA-125: normal; LDH: 286 IU/L↑	Surgery, chemotherapy	DLBCL (non-GCB), II	US: non	CD20(+), CD45(+), Ki-67(+) =40%, CD10(-), bcl-6(-)	NED, 36
21(18)	48y	abdominal pain	weight loss, fatigue	7, left	NM	Surgery, chemotherapy	DLBCL (non-GCB), IV B	US/CT: non	CD20(+), Ki-67(+)>90%, C-Myc(+), PAX-5(+), bcl-2(+), bcl-6(+), MUM-1(+)	NED, 18
22(19)	20y	abdominal pain and distension	No	15, left	CA 125: 202 U/mL↑; LDH: 1714.5 U/L↑; HCG: 47307 mIU/mL↑	Surgery	DLBCL(GCB), III B	US/CT: ovarian retention sign	CD20(+), CD45(+), bcl-6(+), C-Myc(+), PAX-5(+), MUM-1(-)	NED, 3
23	52	abdominal pain	weight loss	14, right	CA 125: 202 U/mL↑; LDH: 1714.5 U/L↑	Surgery, chemotherapy	DLBCL (non-GCB), II B	US/CT/MR/PET: ①ovarian retention sign, ②kissing ovaries, ③vascular floatation, and ④ the sandwich sign	CD19(+), CD20(+), CD45(+), CD79a(+), Bcl-6(+), Bcl-2(+), MUM-1(+)	NED, 24

Abbreviations: CA-125, carbohydrate antigen 125; LDH, lactate dehydrogenase; NM, not mentioned; NED, no evidence of disease.

Table S2. Different characteristics between tumor stage.

	Gross		P value
	Tumor stage I-II	Tumor stage III-IV	
Age (y)[a]	43.8±20.8	41.6±19.7	0.814
B symptoms (n)	11	5	0.242
Diameter (cm) [a]	12.8±4.5	12.9±6.4	0.970
Diameter>10 cm (n) [a]	9	5	0.933
Site (n)			0.066
left	8	3	
right	6	3	
bilateral	0	3	
Elevated level of LDH (n) [b]	8	4	1.000
Elevated level of CA-125 (n) [c]	5	5	0.094
Subtype (n) [d]			0.577
GCB	5	2	
Non-GCB	4	3	
Survival (mo) [e]	17.6±11.0	9.7±7.4	0.150

[a]missing in one patient, [b]missing in eight patients, [c]missing in five patients, [d]missing in nine patients, [e]missing in eight patients

Supplementary References

1. Senol T, Doger E, Kahramanoglu I, Geduk A, Kole E, Yucesoy I, Caliskan E. Five Cases of Non-Hodgkin B-Cell Lymphoma of the Ovary. *Case Reports in Obstetrics and Gynecology* (2014) 2014:1–5. doi: 10.1155/2014/392758

2. Chien JC-W, Chen C-L, Chan WP. Case 210: Primary Ovarian Lymphoma. *Radiology* (2014) 273:306–309. doi: 10.1148/radiol.14121326

3. R B, N K, M M, Rv S. Primary Non-Hodgkin's Lymphoma of the Ovary - A Case Report. *Journal of clinical and diagnostic research : JCDR* (2016) 10: doi: 10.7860/JCDR/2016/19346.7766

4. Pavlovic A, Glavina Durdov M, Lozic D, Skare Librenjak L, Alfirevic D. Primary ovarian lymphoma and benign Brenner tumor. *Taiwanese Journal of Obstetrics and Gynecology* (2016) 55:138–139. doi: 10.1016/j.tjog.2015.04.005

5. Guvvala SL, Sakam S, Niazi M, Skaradinskiy Y. Case of primary bilateral diffuse large B-cell lymphoma of the ovary with plasmablastic features in an HIV-negative female patient. *BMJ Case Reports* (2017)bcr2016218117. doi: 10.1136/bcr-2016-218117

6. P K, P B, M G, M Z, C Y, W H, Hk I, Jt F. Genomic Analysis Reveals Distinct Subtypes in Two Rare Cases of Primary Ovarian Lymphoma. *Pathology, research and practice* (2018) 214: doi: 10.1016/j.prp.2017.12.017

7. Q W, R R, Jz M, L P, D C, D H. Pitfalls of Frozen Section in Gynecological Pathology: A Rare Case of Ovarian Lymphoma in an HIV-Positive Woman Resembling Dysgerminoma on Frozen Section. *International journal of surgical pathology* (2019) 27: doi: 10.1177/1066896918813658

8. M H, A Z, Mf G, M M. Large B cell lymphoma presenting as an adnexal mass in an HIV positive patient: a case report. *The Pan African medical journal* (2019) 33: doi: 10.11604/pamj.2019.33.290.19459

9. Zaidi A, Gupta P, Saha PK, Rajwanshi A, Srinivasan R, Das A. Primary Ovarian Diffuse Large B Cell Lymphoma: Report of a Rare Case in a Young Female. *Journal of Adolescent and Young Adult Oncology* (2019) 8:702–706. doi: 10.1089/jayao.2019.0048

10. N I, Y I, Y F, T S, M O, A K, T O, F K, Y I. Malignant Lymphoma of the Ovary: A Diagnostic Pitfall of Intraoperative Consultation. *International journal of gynecological pathology : official journal of the International Society of Gynecological Pathologists* (2020) 39: doi: 10.1097/PGP.0000000000000571

11. Kaluarachchi A, Bambaranda BgIK, Jayawardena U, De Silva H, Matwani SmA, Rameshkumar U. Ovarian lymphoma in a patient on long-term sulfasalazine for ulcerative colitis. *J Family Med Prim Care* (2020) 9:442. doi: 10.4103/jfmpc.jfmpc_886_19

12. Pc L, Pq L, Yh H, Dc D. Ovarian Diffuse Large B-cell Lymphoma Initially Suspected Dysgerminoma Managed by Laparoscopic Staging Surgery. *Gynecology and minimally invasive therapy* (2020) 9: doi: 10.4103/GMIT.GMIT_79_19

13. Lim YH, Tay AZE, Chew SH, Aggarwal I. Primary ovarian lymphoma. *International Journal of Gynecologic Cancer* (2021) 31: doi: 10.1136/ijgc-2021-003022

14. Sc P, U P, Up S. Primary Unilateral Ovarian Lymphoma in a Young Girl: Case Report. *Journal of obstetrics and gynaecology of India* (2021) 71: doi: 10.1007/s13224-021-01467-0

15. K Ž, I M, A TL, S G, K HP, L S, A C, D H. Torquated large ovarian lymphoma as a cause of acute abdomen: a case report of diffuse large B-cell ovarian lymphoma with a germinal center B-cell-like phenotype. *Wiener medizinische Wochenschrift (1946)* (2022) 172: doi: 10.1007/s10354-021-00823-z

16. Luo B, He R, Peng Z, Ma J, Feng Z, Chen G, Zeng J. Unusual presentation of primary ovarian diffuse large B-cell lymphoma: a case report. *J Ovarian Res* (2022) 15:47. doi: 10.1186/s13048-022-00978-2

17. Jaouani L, Zaimi A, Al Jarroudi O, Berhili S, Brahmi SA, Afqir S. An Extranodal Site of Diffuse Large B-cell Lymphoma Presenting as Ovarian Cancer. *Cureus* (2023) doi: 10.7759/cureus.34337

18. M U, K N, A S, T P. A Rare Case of Ovarian Double-Hit/Diffuse Large B-Cell Lymphoma: A Case Report and Review of Literature. *Journal of investigative medicine high impact case reports* (2023) 11: doi: 10.1177/23247096231154641

19. Gerrity C, Mercadel A, Alghamdi A, Huang M. Primary ovarian lymphoma: A case report. *Gynecologic Oncology Reports* (2023) 47:101212. doi: 10.1016/j.gore.2023.101212
